# Biology of mitral valve prolapse: from general mechanisms to advanced molecular patterns—a narrative review

**DOI:** 10.3389/fcvm.2023.1128195

**Published:** 2023-06-02

**Authors:** Daniele Ronco, Gianpiero Buttiglione, Andrea Garatti, Alessandro Parolari

**Affiliations:** ^1^ Department of Congenital Cardiac Surgery, IRCCS Policlinico San Donato, Milan, Italy; ^2^ Department of Universitary Cardiac Surgery, IRCCS Policlinico San Donato, Milan, Italy; ^3^ Department of Cardiothoracic Surgery, Heart and Vascular Centre, Maastricht University Medical Centre, Maastricht, Netherlands; ^4^ Department of Biomedical Sciences for Health, University of Milan, Milan, Italy

**Keywords:** mitral valve prolapse, mitral regurgitation, molecular biology, fibroelastic deficiency, Barlow disease, Marfan syndrome, Loeys-Dietz syndrome, Ehlers-Danlos syndrome

## Abstract

Mitral valve prolapse (MVP) represents the most frequent cause of primary mitral regurgitation. For several years, biological mechanisms underlying this condition attracted the attention of investigators, trying to identify the pathways responsible for such a peculiar condition. In the last ten years, cardiovascular research has moved from general biological mechanisms to altered molecular pathways activation. Overexpression of TGF-β signaling, for instance, was shown to play a key role in MVP, while angiotensin-II receptor blockade was found to limit MVP progression by acting on the same signaling pathway. Concerning extracellular matrix organization, the increased valvular interstitial cells density and dysregulated production of catalytic enzymes (matrix metalloproteinases above all) altering the homeostasis between collagen, elastin and proteoglycan components, have been shown to possibly provide a mechanistic basis contributing to the myxomatous MVP phenotype. Moreover, it has been observed that high levels of osteoprotegerin may contribute to the pathogenesis of MVP by increasing collagen deposition in degenerated mitral leaflets. Although MVP is believed to represent the result of multiple genetic pathways alterations, it is important to distinguish between syndromic and non-syndromic conditions. In the first case, such as in Marfan syndrome, the role of specific genes has been clearly identified, while in the latter a progressively increasing number of genetic loci have been thoroughly investigated. Moreover, genomics is gaining more interest as potential disease-causing genes and loci possibly associated with MVP progression and severity have been identified. Animal models could be of help in better understanding the molecular basis of MVP, possibly providing sufficient information to tackle specific mechanisms aimed at slowing down MVP progression, therefore developing non-surgical therapies impacting on the natural history of this condition. Although continuous progress has been made in this field, further translational studies are advocated to improve our knowledge of biological mechanisms underlying MVP development and progression.

## 1. Introduction

Mitral valve prolapse (MVP) represents the most common cause of moderate to severe primary mitral regurgitation (MR), affecting 0.6%–3.0% of the general population, and it is in fact the leading indication for isolated MV surgery in most developed countries (1). Generally, it is defined as diffuse redundancy or thickening of the MV leaflets with systolic motion of one or both leaflets beyond the plane of the MV annulus, with or without associated MR (2, 3). Nevertheless, such definition includes a wide variety of specific conditions, since MVP patients may present heterogeneous clinical and anatomical characteristics (2, 4). Similarly, the potential biological mechanisms underlying the development of MVP may be either sporadic or genetically inherited (5).

The translational research on MVP has traditionally started from its peculiar histopathological characteristics, eventually moving into pathophysiological processes involved in the onset of MVP and ultimately into MR progression, including oxidative stress, hemostatic alterations, platelet malfunctioning and, most of all, extracellular matrix (ECM) remodeling (4, 6). Currently, the focus of MVP research has shifted to the identification of altered molecular pathways activation and of the underlying genetic abnormalities associated with those pathways (5).

A pivotal role in the etiopathogenesis of the myxomatous phenotype of MVP is played by altered transforming growth factor-β (TGF-β) signaling pathway, that can however be somehow limited with the help of angiotensin-II inhibitors (5, 7, 8). Nonetheless, other molecular pathways and genetic alterations have been observed in different phenotypes of the MVP spectrum (9). However, it is also important to underline that most of the evidence on MVP biology and molecular patterns comes from animal studies, mainly mouse models, and have been barely carried out in humans.

Mitral valve prolapse may present as either a sporadic form of primary degenerative MV disease or in familial clusters where some genetic patterns can possibly be involved (10). Either form of MVP has peculiar characteristics, both in the clinical course and in the histopathologic presentation. Indeed, while the familial, inherited forms of MVP present more frequently as a Barlow disease, with abundant, myxomatous leaflets, the sporadic, degenerative forms generally show the features of fibroelastic deficiency (4). Finally, MVP may present as a component of the cardiac alterations involving a significant proportion of patients affected by connective tissue diseases (CTDs), such as Marfan syndrome, Loeys-Dietz syndrome (LDS), Ehlers-Danlos syndrome (EDS) and pseudoxanthoma elasticum (PXE) (11). In the syndromic forms of MVP, the pattern of the disease is more clearly reproducible and specific genetic patterns have been thoroughly identified, with some of them specifically weighing more in the pathogenesis of MVP (9). The main differences between non-syndromic and syndromic forms of MVP are presented in Table 1.

**Table 1:** Main characteristics distinguishing non-syndromic and syndromic mitral valve prolapse.

Characteristics	Non-syndromic MVP	Syndromic MVP
Hereditary transmission	Sporadic or familial forms	Defined genetic transmission from known syndromes (most often CTDs)
Prevalence	More common (about 2% of general population)	Less common (1:5,000 births)
Age at diagnosis	Middle aged adults	Young age/at birth
Clinical manifestation	Isolated, primary mitral regurgitation	Mitral regurgitation associated with other organs involvement according to the specific syndrome
Mutations	Multifactorial genetic alterations and external factors	Specific gene mutations
Molecular pathways involved	TGF-β pathway activation Upregulation of adherence molecules	TGF-β pathway overexpression Nuclear accumulation of SMAD2
Histological phenotypes	Accumulation of myxoid ECM in leaflets and chordae tendineae	Proliferation of valvular interstitial cells Defect in collagen fibers Calcium accumulation

CTD, connective tissue disease; ECM, extracellular matrix; TGF-β, transforming growth factor-beta.

We performed a narrative review to present the main features of MVP biology, from the genetics and embryology to the pathophysiological processes involved in the development of this condition, as they have been identified throughout the years, mostly on animal models.

## 2. Gross anatomy of mitral valve

The MV separates the left atrium from the left ventricle (LV). It is characterized by a complex composed of a valvular and sub-valvular apparatus. The valvular apparatus is mainly represented by two leaflets, anterior and posterior, which insert on an elliptical, saddle-shaped fibrous ring that is part of the fibrous skeleton of the heart (4). The anterior leaflet generally covers a significantly larger portion of the MV area, while the posterior one lines almost two-thirds of the mitral annulus. The latter also presents a few small notches on its free margins from which it is possible to ideally divide the leaflet into three scallops that are counterclockwise labeled as P1, P2 and P3; from them, we can also identify the corresponding scallops on the facing anterior leaflet (i.e., A1, A2 and A3). The two mitral leaflets are connected to each other through the anterolateral and the posteromedial commissures (4).

The sub-valvular apparatus is composed by the chordae tendineae that can be primary (marginal), secondary (intermediate), or tertiary (basal) and connect the mitral leaflets to generally two papillary muscles, namely the anterolateral and posteromedial ones (4). An integral component of the sub-valvular apparatus is represented by the LV itself, which plays an active role in the proper functioning of the MV. Indeed, the functional competence of the MV is provided by the mitral-ventricular complex, where all those structures actively contribute to the proper opening and closure of the valve. For instance, also the annulus actively facilitates valve competency by contracting during systole, reducing MV orifice area by 20%–30%, and slightly displacing towards the LV apex.

Within the heart, the MV is also connected with the remaining of the fibrous skeleton of the heart at the level of the aorto-mitral curtain, in continuity to the anterior portion of the MV annulus.

### 2.1. Embryology of mitral valve

The MV forms between the 5th and the 8th weeks of gestation (12). During early morphogenesis, in the atrioventricular canal the cardiac cushions develop from a regional swelling of the ECM, populated by mesenchymal cells derived from endothelial cells previously undergoing endothelial-to-mesenchymal transition, thus forming the primordial valve which separates the cardiac chambers (13). This process is regulated by bone morphogenic protein (BMP), a cytokine belonging to the large group of TGF-β. During fetal life, BMP-2, produced in the myocardium, and BMP-4 play an important role to initiate the endothelial-to-mesenchymal transition (14). Moreover, other proteins, such as Wnt/β-catenin, hippo/yap, and Notch, promote the development of the endocardial cushions though their signaling pathways. Endothelial-to-mesenchymal transition is activated by SRY-box transcription factor-9 (SOX9) that, when lacking, makes mesenchymal cells unable to promote cardiac cushion cells proliferation through Erb-B2 receptor tyrosine kinase 3 (ERBB3) enzyme expression (15).

Progressing throughout fetal heart development, the interaction between cell proliferation and ECM organization promotes the elongation and stratification of the primordial valve into the mature valve leaflets. During this stage, vascular endothelial growth factor stimulates the proliferation of valvular endothelial cells.

The valvular interstitial cells, a specialized subpopulation of mesenchymal cells already present in the primordial valve, differentiate to create collagen-rich and elastin-rich ECM layers (16, 17). The first transcription factors are activated by calcineurin signaling; subsequently, embryological valve maturation is promoted by Wnt/β-catenin signaling, fibroblast growth factor-4, SOX4, and other downstream modulators of the TGF-β superfamily pathways, including Mothers against decapentaplegic homolog 6 (SMAD6) (16). Mutations affecting each of these pathways may eventually result in a structural or functional alteration of the MV.

In the postnatal period, the MV undergoes a final remodeling leading to the mature form with the three-layered structure that is described below (18).

### 2.2. Histology of mitral valve

**Figure 1:**
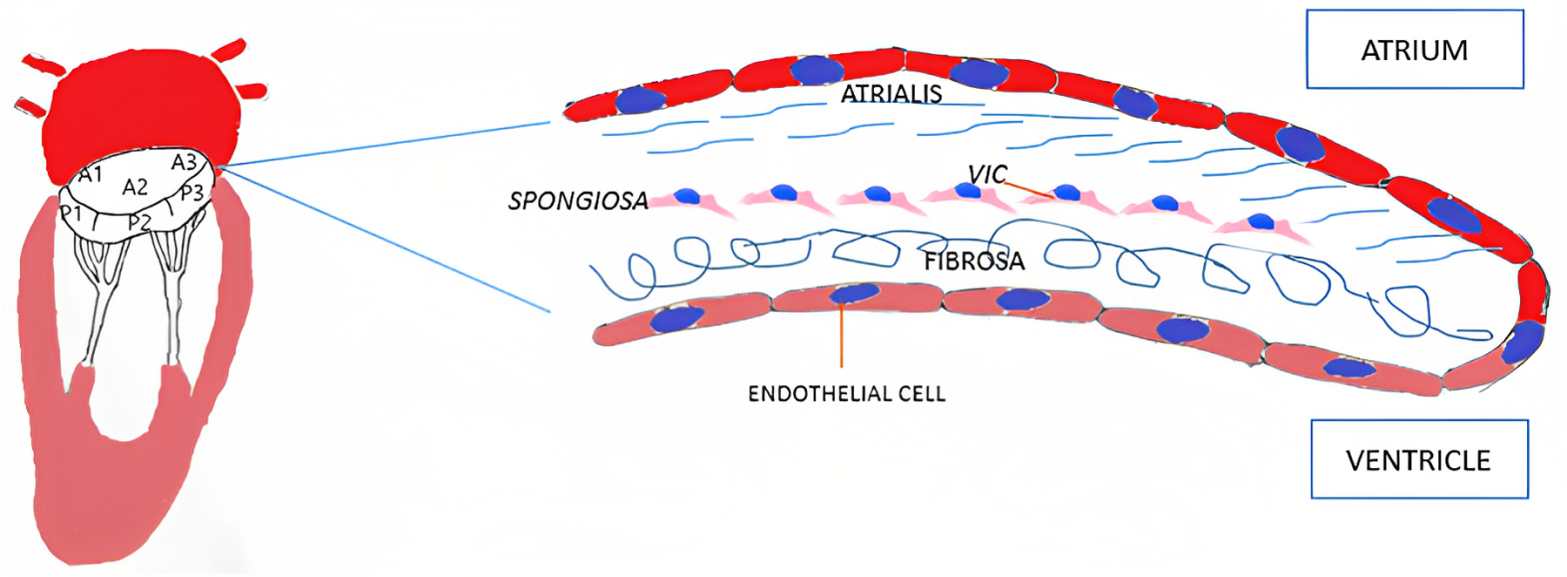
Mitral valve histology. VIC, valvular interstitial cell.

From a histological point of view, MV leaflets are organized into three layers: atrialis, spongiosa and fibrosa (Figure 1). The first layer is constituted of lamellar collagen fibers and elastin sheets that extend from the left atrial endocardium into the leaflet reaching the free margin. On the ventricular side of the valve, the fibrosa is found, a layer made of dense collagen fibers that extends from the mitral annulus downwards. The fibrosa is thicker in proximity to the MV annulus and gets thinner towards the free margins of the leaflets (19). Between the two layers the spongiosa is located, a loose connective tissue layer rich in glycosaminoglycans. Differently from the previous one, this layer is thicker at the leaflet edge and thinner towards the annulus.

Mitral leaflets are layered by endothelial cells on both sides. The underlying subendothelial layer contains quiescent valvular interstitial cells, noncontractile, fibroblast-like cells of endocardial origin that contribute to the homeostatic remodeling of the ECM components (20). The MV leaflets are connected to the chordae tendineae that are made of a collagenous cylindrical structure embedded within an elastin sheath (4, 21).

### 2.3. Common histological phenotypes of mitral valve prolapse

The peculiar histological alterations associated with MVP most often represent the result of altered TGF-β signaling or related proteins (3). In myxomatous MVP the leaflets tend to present a higher cell density, especially at the level of the spongiosa, which shows a significantly increased thickness, varying also with the level of accumulation of myxoid ECM rich in glycosaminoglycans and with the degree of fibrosis in the atrialis layer (6). Such myxomatous ECM is also responsible for the progressive disruption of the fibrosa. Indeed, in both the leaflets and chordae tendineae this altered, excessive ECM replaces much of the collagen fibers and elastin integrity (22). Typically, chordae tendineae tend to present more water and less collagen content, contributing to the reduced tensile strength observed in MVP (22). Expansion of the spongiosa is paralleled by plaque formation that represents the main responsible of the thickened aspect of MV leaflets in myxomatous degeneration. In this context, valvular interstitial cells undergo activation towards a myofibroblast-like phenotype, characterized by the expression of vimentin and some other smooth muscle proteins, but not smooth myosin isoforms 1 and 2 (SM1 and SM2) that are typical of differentiated smooth muscle cells (5). Moreover, in myxomatous MV leaflets it is possible to identify the presence of CD45-positive cells, representing fibrocytes able to further differentiate into myofibroblasts, serving the same function as activated valvular interstitial cells (5). It is noteworthy that some syndromic forms of MVP present peculiar histological patterns derived from the underlying defect (e.g., pseudoxanthoma elasticum), which will be discussed later in this paper (23).

## 3. Non-syndromic forms of mitral valve prolapse

In forms not associated with CTDs or related syndromes, MVP presents sporadically in the majority of cases, albeit sometimes it is possible to identify some familial clusters. Moreover, from the clinical and histopathological points of view, MVP may be classified as either fibroelastic deficiency or Barlow disease, which represent two very distinctive forms of this condition (24).

### 3.1. Fibroelastic deficiency

Fibroelastic deficiency is characterized by thin and translucent leaflets deficient in collagen, elastin, and proteoglycans, with occasionally some excess tissue and few calcifications (22, 24). The prolapse involves a few leaflet segments, usually P2 only, and chordae tendineae are thin and frequently ruptured. MV annulus is mildly enlarged as a result of a MR that often progresses rapidly (within 5 years) (24). In fibroelastic deficiency, it has been observed that the MV annulus is characterized by a strong anteroposterior contraction and saddle-shape accentuation during early systole, followed by a lack of annular expansion later in systole (25). This condition is more frequently encountered in patients aged 60 years or more.

### 3.2. Myxomatous mitral degeneration and Barlow disease

**Figure 2:**
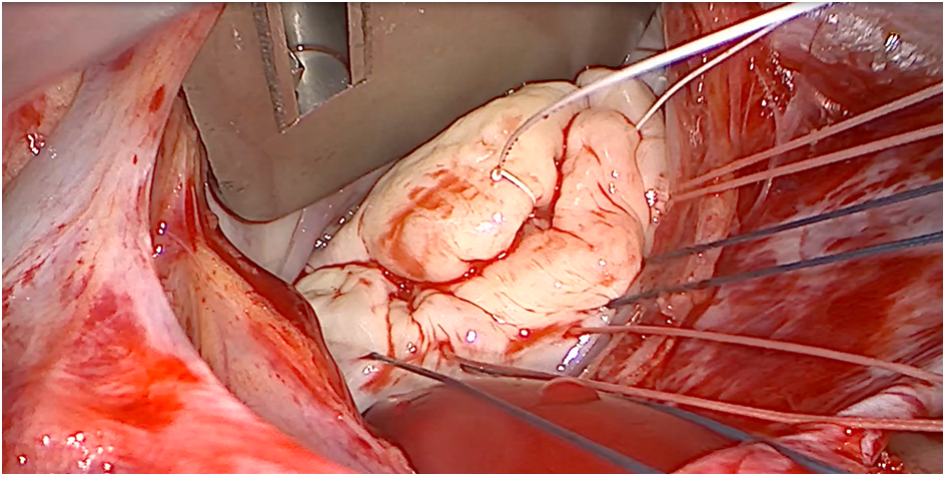
Intraoperative picture of non-syndromic Barlow disease, showing bi-leaflet prolapse with thickened, redundant valvular tissue and elongated chordae tendineae, resulting from myxoid extracellular matrix accumulation.

Barlow disease is characterized by thickened, redundant leaflets as the result of a diffuse, excessive proliferation of connective tissue (Figure 2) (24). It generally develops slowly along the years and is also associated with severe annular dilatation, irregular, abundant and elongated chordae tendineae, sometimes ruptured, and considerable tissues calcification (24). Histologically, it is possible to observe disrupted collagen and elastic layers, and excessive proteoglycans accumulation (22). In this condition, early systole is characterized by a reduced annular contraction and saddle-shape accentuation, while late systolic phase shows an excessive annular expansion (25). This type of myxomatous MVP often leads to multi-segmental MR before the age of 60 and is typical of the familial forms.

### 3.3. Genetic alterations of non-syndromic mitral valve prolapse

Although not recognized in most contexts, because clinically silent, and apparently presenting mostly in isolated cases, familial forms of myxomatous MVP have been observed. In fact, some studies reported a familial MVP transmission in as much as 60% of patients (10). They involve multiple, complex genetic pathways mostly associated with tissue strength and ECM remodeling, following either an autosomal dominant or an X-linked pattern of transmission with reduced, sex- and age-dependent penetrance. The main genes associated with non-syndromic form of MVP, as well as the molecular pathways directly affected are presented in Table 2. Moreover, the spectrum of MV alterations in familial clusters of MVP may present early disease expression in gene carriers, whereby allowing to anticipate disease progression and possibly try to influence its clinical course.

**Table 2:** Summary of main genetic and molecular pathway alterations associated with non-syndromic mitral valve prolapse.

Occurrence	Genes	Proteins	Function
**Familial**	**Autosomal dominant**		
	- *MMVP1*	–	–
	- *MMVP2*	–	–
	- *MMVP3*	–	–
	- *DCHS1*	- Protein dachsous homolog 1	- Cadherin: cell-to-cell adhesion for tissue morphogenesis and homeostasis
	**X-linked**		
	- *FLNA*	- Filamin A	- MAPK3 and MAP2K1 activation for mitral valve tissue proliferation
**Sporadic**	- *DCHS1*	- Protein dachsous homolog 1	- Cadherin: cell-to-cell adhesion for tissue morphogenesis and homeostasis
	- *DZIP1*	- DAZ interacting Zinc finger protein	- Cilium assembly
	- *FLNC*	- Filamin C	- Cell-to-cell adhesion
	- *MMP-3*	- Matrix metalloproteinase-3	- ECM remodeling
	- *FBN1*	- Fibrillin-1	- Decrease binding of TGF-β binding proteins
	- *COL3A1*	- Collagen type III α1 chain	- ECM structural protein for tissue resistance
	- *LIM*	- Protein interaction domain	- Cell migration regulation
	- *LMCD1*	- LIM and cysteine-rich domains protein	- Cell migration regulation
	- *TNS1*	- Tensin 1	- Cell adhesion control
	- *GLIS1*	- Glis family Zinc finger 1	- Transcription factor involved in cell reprogramming and proliferation
	- *LTBP2*	- Latent TGF-b binding protein 2	- ECM protein
	- *TGFB2*	- TGF-b 2	- ECM protein
	- *ALPK3*	- Alpha kinase 3	- Myocyte hypertrophy

ECM, extracellular matrix; TGF-β, transforming growth factor-beta.

#### 3.3.1. Autosomal inheritance

Familial inheritance of MVP in patients without the features of CTDs or related syndromes is known for decades (10, 26). However, the genetic basis of this disease and its molecular implications in the development of MVP are still poorly understood (9). Indeed, in addition to the X-linked forms of MVP, autosomal inheritance is more common, but traditionally only three loci have been identified as associated with familial MVP, namely chromosome 16p11.2-p12.1 [Myxomatous MVP-1 (*MMVP1*), the first locus identified in 1999], 11p15.4 [Myxomatous MVP-2 (*MMVP2*), also identified as a potential disease-causing gene], and 13q31.3-q32.1 [Myxomatous MVP-3 (*MMVP3*)] (9, 15, 26, 27). Furthermore, polymorphisms and epigenetic alterations known to be deleterious in the Dachsous cadherin-related 1 (*DCHS1*) gene encoding for a member of the cadherin proteins (mainly involved in protein stability) have been associated with familial and sporadic forms of MVP (27, 28). This gene, as well as DAZ interacting Zinc finger protein 1 (*DZIP1*), is involved in primary cilia biology that has been shown to play a role in MVP development, including ECM expansion that is observed in myxomatous valve alterations (27).

Filamin C gene mutations leading to weakened cell-cell adhesion have also been identified to potentially cause a peculiar arrhythmogenic MVP syndrome (27).

A polymorphism of the matrix metalloproteinase-3 (*MMP-3*) gene has been shown to affect MV disease severity, possibly serving as a marker of adverse and rapid clinical course. Similarly, other polymorphisms in genes involved in ECM remodeling, collagen metabolism, and other pathophysiological pathways have been observed in sporadic cases of MVP (22). Examples include the association between MVP onset and progression, and certain genotypes of fibrillin-1 gene (*FBN1*) and *COL3A1* gene (encoding for collagen type III α1) that possibly make fibrillin-1 and collagen less resistant and more extensible, similarly to other well-known CTDs (29).

Other loci possibly associated with the development of MVP have been identified on chromosomes 2q35, 17p13, 22q12, and 1p32.3 (9, 27) The first of these loci includes two genes found to be involved in mitral and tricuspid valves regurgitation, namely LIM and cysteine-rich domains protein 1 (*LMCD1*), regulating cell migration and replication, and Tensin 1 (*TNS1*), involved in focal adhesion control (27, 30). On chromosome 1, the Glis family Zinc finger 1 (*GLIS1*) gene is located, which is usually expressed on MV endothelial and mesenchymal cells in zebrafish models and whose deletion might play a pathogenetic role in MVP (27, 31).

#### 3.3.2. X-linked inheritance

Concerning the smaller group of familial MVPs that don't have autosomal inheritance, the X-linked myxomatous MV dystrophy represents a rare condition related to mutations involving chromosome Xq28 locus (27). Such inherited disorder has been shown to be associated with Filamin A (*FLNA*) gene mutation, which follows a sex-related different penetrance, incomplete for women and complete for men, respectively (27, 32). Thus, MVP phenotype involving *FLNA* mutations is now accepted to affect both degenerative and congenital alterations of MV structure.

#### 3.3.3. Genome-wide association studies

Although knowledge about the underlying genetic mechanisms leading to MVP is still limited and their relevance often remains unclear in sporadic forms, as mentioned above, a recent meta-analysis of six genome-wide association studies including almost 5,000 MVP patients was able to identify 14 genetic loci related to MVP (including *LMCD1* and *TNS1*) and to develop a polygenic risk score potentially able to stratify patients according to their genetic risk profile for this condition (9). Moreover, such analysis strengthened the genetic evidence of the role of TGF-β signaling in MVP development, as previously suggested mostly for the syndromic forms of the disease. Indeed, among the relevant loci, two of them are directly involved in such pathway, namely latent TGF-β binding protein 2 (*LTBP2*) and TGF-β2 (*TGFB2*) genes (9). *LTBP2* encodes for an ECM protein associated with fibrillin-1 and it Is involved in TGF-β signaling and in the pathogenesis of some CTDs (9, 33). Among the other genes potentially implicated in MVP development there is alpha-protein kinase 3 (*ALPK3*), which is also associated with non-sarcomeric hypertrophic cardiomyopathy, therefore potentially suggesting an overlap between the myopathic and the valvular pathogenic pathways observed in this condition, where often MV disease is considered a primary phenotypic expression of the disease, together with myocyte hypertrophy (9, 34).

Although various genetic and molecular alterations have been associated with this pleiomorphic condition along the years, both in sporadic, familial, and syndromic forms, it is noteworthy that no biomarker for MVP has been clearly identified yet, nor a molecule has been validated for its potential predictive role in clinical setting.

### 3.4. Molecular pathways involved in MVP pathogenesis

**Figure 3:**
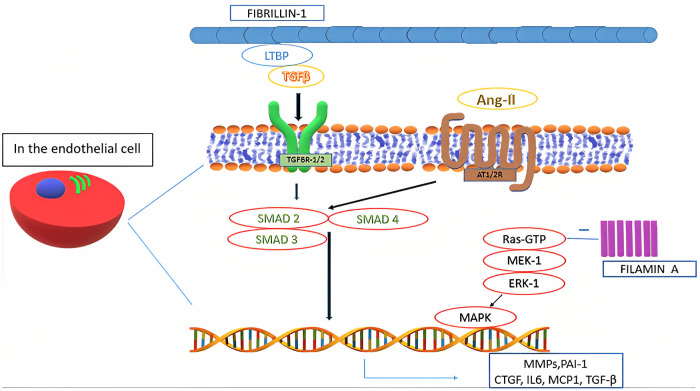
Biological mechanisms involved in mitral valve prolapse.

In the pathogenesis of MVP all the components of MV tissue are affected. Moreover, even in non-syndromic MVP, a complex, multifactorial interplay of genetic alterations and external factors, as well as mechanical stress, contributes to the development of alterations typical of myxomatous MVP, including TGF-β1 pathway activation and impaired expression of adherence molecules (e.g., upregulation of cadherin-11, vimentin and β-catenin, and downregulation of N-cadherin), transcription factors, and MMPs (Figure 3) (6, 29). Such altered expression by valvular interstitial cells has also been demonstrated to correlate with disease severity. Indeed, in the myxomatous MV degeneration, activation and proliferation of valvular interstitial cells that further differentiate into myofibroblasts (expressing vimentin and α-smooth muscle actin) are responsible for TGF-β pathway upregulation (considered the key player in the myxomatous MV) and catabolic enzymes expression which, together with possible mutations in cytoskeletal proteins (e.g., filamins), lead to a significant ECM reorganization through an increased production and turnover (6, 15, 35). Moreover, TGF-β further promotes cell proliferation and myofibroblasts differentiation (4).

As a result, collagen degradation and elastin fragmentation by upregulated catalytic enzymes, especially collagenases and gelatinases (e.g., MMP-1, MMP-2, MMP-3, MMP-9, MMP-13, cathepsin S and cathepsin K), and a substantial increase in the proteoglycan component determine the new altered ECM typical of the myxomatous MV phenotype (3, 6, 29). Such new histological and clinical configuration further increases the mechanical stress on the valvular interstitial cells, therefore perpetuating the pathophysiological mechanisms associated with this condition (6, 36). Furthermore, higher levels of reactive oxygen species have been documented in MVP patients as compared to subjects with coronary artery disease and with aortic valve stenosis (29).

In the pathogenesis of MVP, also a significant crosstalk between valvular interstitial cells and valvular endothelial cells has been observed in various studies (37). Physiologically, such interaction is known to regulate cellular homeostasis, by inhibiting valvular endothelial cells endothelial-to-mesenchymal transition and valvular interstitial cells activation (6, 37). However, endothelial-to-mesenchymal transition has been shown to occur extensively and to be involved in MVP pathogenesis (6). Such differentiation also induces the expression and secretion of osteoprotegerin, a soluble glycoprotein belonging to the tumor necrosis factor family with the role to regulate bone metabolism in the osteoblast-osteoclast interaction (38). Osteoprotegerin has also been shown to be involved in coronary artery diseases and aortic valve stenosis. Interestingly, this protein has been observed to exacerbate MVP phenotype, being involved in its pathogenesis via various direct and indirect mechanisms, namely: induction of reactive oxygen species; endothelial cells migration with increased valvular endothelial cells endothelial-to-mesenchymal transition (also overexpressing BMP-4); collagen production; MMP-9 overexpression; valvular interstitial cells proliferation; and proteoglycans overexpression (38). Moreover, osteoprotegerin is involved in vascular remodeling together with cathepsin K, which has also an extensive role in ECM remodeling (38). Furthermore, it has been suggested that high circulating plasma levels of osteoprotegerin may selectively identify MVP patients, although lacking in specificity (38).

Concerning ECM reorganization, a significant increase in hyaluronic acid content with reduced sulphated glycosaminoglycans may be observed (22). Such alteration can be related to the TGF-β-induced downregulation of genes belonging to the A disintegrin and metalloproteinase with thrombospondin motifs (*ADAMTS*) family that play a central role in proteoglycans degradation (5, 29). Thus, it may be responsible for the altered mechanical properties of myxomatous MV leaflets, leading to an increased valve extensibility, paralleled by a decrease in leaflet stiffness and failure strain (5). Moreover, it has been observed that areas of chordal rupture tend to show increased expression of vascular endothelial growth factor-1 and absence of tenomodulin, demonstrating altered angiogenic processes in myxomatous valves (5, 29). Furthermore, higher numbers of inflammatory cells expressing high levels of MMP-2 and MMP-13 can be found in the same context. Increased MMPs levels have also been found in atrial appendage specimens of patients undergoing MV surgery, hence suggesting an extended organ involvement of certain histological alterations typical of MVP (5, 29). Moreover, some functional single nucleotides polymorphisms of certain *MMPs* (especially *MMP-2* and *MMP-9*) have been documented in patients with MVP and may correlate with disease severity (5). A mutation in fibrillin-1 may decrease local binding of TGF-β large latent complex. Moreover, TGF-β binding to its receptors (TGFBR1/2) activates multiple signaling pathways. Such activation increases the nuclear transcription of several proteins, including TGF-β itself. An interaction between TGF-β and angiotensin-II receptors type 1 and 2 (AT1R and AT2R) has also been identified and it represents a possible therapeutic target through selective AT1R antagonism (39). Moreover, certain polymorphisms affecting *AT1R* have been associated with an increased risk of occurrence and progression of MVP. On the other side, SMAD2/3 and p38 have been shown to stimulate TGF-β-induced ECM production by valvular interstitial cells (5).

Filamin A deficiency may also be associated with MV tissue proliferation through the excessive activation of the mitogen-activated protein kinase 3 (MAPK3) and mitogen-activated protein kinase kinase 1 (MAP2K1) signaling pathways (32, 40).

An osteoblastic cell differentiation mediated by the low-density lipoprotein receptor-related protein 5 signaling pathway has also been associated with progressive leaflet thickening in MVP (41, 42). More typically, mitral leaflet tissue in a prolapsing valve displays a phenotypic expression of the endochondral bone differentiation resembling cartilage tissue, which is consistent with the gross appearance of MVP leaflets. Such differentiation potential of the valvular endothelial cells may suggest their physiological ability to generate valvular interstitial cells present in specific regions of the MV, supporting the valve self-renewal as well (5, 17, 36). These processes have been shown to be activated during MVP pathogenesis, possibly also reflecting a stress response mechanism.

### 3.5. Concomitant pathophysiological alterations

Various pathophysiological processes have been studied for their possible involvement in MVP. However, most available evidence has been drawn from animal studies, mostly from mouse and zebrafish models, and found poor confirmation in humans (29).

Although contrasting data are available in literature, a link between MVP, especially when clinically relevant, and alterations in the hemostatic pathways, sometimes leading to a pro-thrombotic state associated with higher rates of thromboembolic events, has been observed and discussed since the 80s (43). However, a consensus on the real hemostatic alterations present in this condition has not been reached yet. Moreover, altered platelet function has also been observed, together with an increased thrombin generation (29, 44). Hemostatic perturbations and platelet activation have been observed to be related to the degree of MR, therefore supporting the hypothesis that such alterations represent a consequence of the hemodynamic alterations caused by MV disease (29). As a matter of fact, secondary shear activation and aggregation of platelets due to blood regurgitation through diseased leaflets seems also to explain the platelet dysfunction observed in valvular heart diseases, such as MVP (44). Nevertheless, such observations have been mostly emerged in canine models and few data exist to confirm the link between MVP and specific alterations in the hemostatic pathways (29).

The role of oxidative stress in MVP has also been explored. Indeed, an increased oxidative stress is often found in patients with valvular heart diseases, including MR (29). Particularly, animal studies showed both systemically and locally increased nitric oxide synthase (NOS) expression in leaflet areas showing myxomatous degeneration, therefore suggesting a possible role of nitric oxide in MVP pathogenesis (45). Moreover, endothelial dysfunction has also been observed and it is possibly driven by the degree of MR (29, 45).

An impairment in the renin-angiotensin-aldosterone system (RAAS) has also been identified in the early phases of MVP (29). Indeed, all the components of this pathway appear to be increased in dosage or activity, with still normal levels of endothelin-1, atrial natriuretic peptide, and arginine vasopressin (i.e., markers of congestive heart failure) (29). Thus, it seems that RAAS overactivation represents a consequence of the MV disease itself and not, instead, the result of heart failure that may develop thereafter (29).

Finally, it has been observed that electrolyte imbalance, especially manifested by hypomagnesemia (mirroring low total intracellular erythrocyte magnesium), and autonomic dysregulation, such as increased α-adrenergic status and excessive vagal tone, may play a role in MVP, potentially representing also therapeutic targets to partially control secondary symptoms associated with this condition (29).

## 4. Syndromic forms of mitral valve prolapse

**Figure 4:**
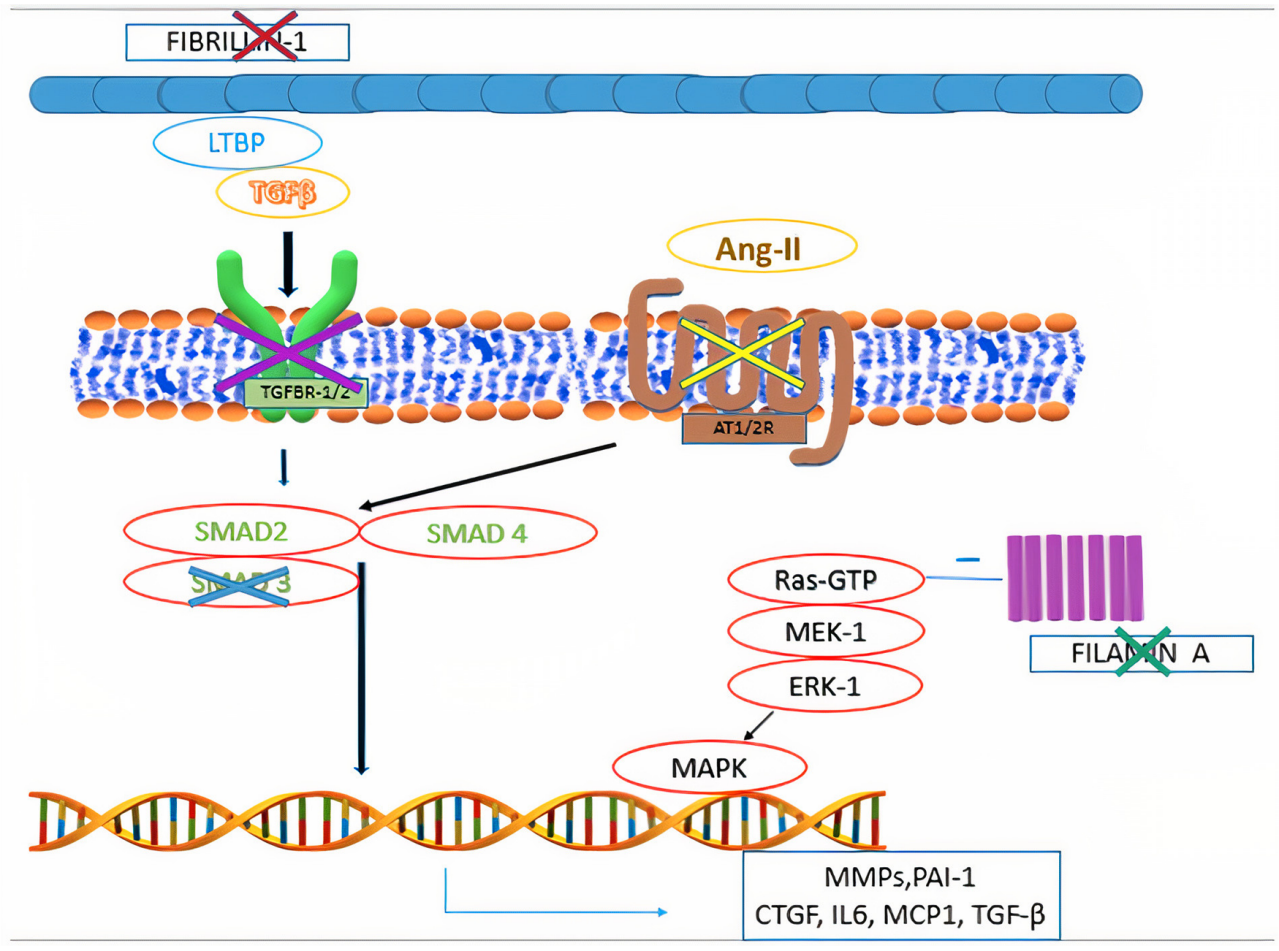
Main pathophysiological mechanisms identified in syndromic mitral valve prolapse. Mutations in fibrillin-1 gene are associated with Marfan syndrome. Mutations in filamin A are associated with Filamin A mutation syndrome. Mutations in the genes encoding for components of the transforming growth factor-β receptors 1 and 2 (TGFBR1 and TGFBR2) are associated with Loeys-Dietz syndrome. Mutations in *MADH3*, which encodes for SMAD3, are associated with aneurysms-osteoarthritis syndrome. Selective angiotensin-II receptor type 1 (AT1R) antagonism (e.g., Losartan) is associated with decreased levels of SMAD2, providing potential therapeutic benefits to subjects with fibrillin-1 mutation.

Mitral valve prolapse may frequently occur also in inherited CTDs, where TGF-β dysregulation has been shown to play a central role (Figure 4). These conditions include Marfan syndrome, Loeys-Dietz syndrome, Ehlers-Danlos syndrome, pseudoxanthoma elasticum and aneurysms-osteoarthritis syndrome (11). Moreover, other disorders, such as filamin A mutation syndrome and osteogenesis imperfecta, have been characterized by higher rates of MVP, as well. Among other inherited syndromes frequently presenting with MVP, adult polycystic kidney disease has been reported to show this condition in about 25% of the patients (46). A much rarer condition, Williams syndrome, may cause MVP secondary to elastin gene mutation (47). The genetic and molecular peculiarities of the main syndromes associated with MVP are presenting in Table 3.

**Table 3:** Summary of main genetic and molecular pathway alterations associated with syndromic mitral valve prolapse.

Clinical syndromes	Genes	Proteins	Functions
Marfan syndrome	- *FBN1*	- Fibrillin-1	Structural and non-structural functions of connective tissue
	*-FBN2*	-Fibrillin-2	
	- *TGFB2*	- TGF-β2	
Loeys-Dietz syndrome	- *TGFBR1*	- TGF-β receptor 1	Excessive TGF-β signaling: SMAD2 accumulation increasing connective tissue formation
	- *TGFBR2*	- TGF-β receptor 2	
Aneurysms-osteoarthritis syndrome	- *MADH3*	- SMAD3	TGF-β overexpression
Ehlers-Danlos syndrome	- *COL1A2*	- Collagen type I α2 chain	Defective collagen fibers in connective tissue
Pseudoxanthoma elasticum	- *ABCC6*	- MRP6	Lack of function: calcium deposits in elastic fibers
Filamin A mutation syndrome	- *FLNA*	- Filamin A	Embryological development, cell migration and response to mechanical stress

TGF-β, transforming growth factor-beta.

### 4.1. Marfan syndrome

Marfan syndrome is an autosomal dominant CTD that affects 1 in 5,000 patients (48). This condition is associated with ocular, musculoskeletal and cardiovascular alterations that may significantly impact on patients' quality of life and life expectancy (49, 50). The first diagnosis is clinical, according to the Ghent criteria (in fact also including the presence of concomitant MVP), and eventually confirmed by genetic and molecular tests (51). Concerning the cardiovascular system involvement, Marfan syndrome is often characterized by diffuse aortic pathology, including aortic aneurysm and dissection (52). However, the majority of patients with Marfan syndrome is diagnosed with some form of MVP, as well (median 56.7%) (27, 53). Indeed, it has been observed that the probability of developing MVP increases to 77% and the risk of developing MR approximates 60% by the age of 60 years in Marfan syndrome patients. Nonetheless, one-tenth of them will undergo surgery due to MR (15).

Marfan syndrome represents the result of mutations in the *FBN1* gene (chromosome 15q15-q21) and of inactivating mutations in the *TGF-*β*2* and its receptor (*TGFBR2*), whose genes are located on chromosome 3p24.2-p25 (25,51,52). Although *FBN1* mutations play a central role in the clinical manifestations of Marfan syndrome, fibrillin-2 (*FBN2*) mutations have also been shown to potentially cause the disease (54). Fibrillins are high-molecular weight ECM proteins serving both structural and non-structural functions. Structurally, fibrillin proteins represent the main component of ECM microfibrils that provide mechanical and elastic support to the connective tissue (55). Moreover, these proteins are also implicated in the cell signaling regulation, similarly to the LTBPs (54). It is noteworthy that Marfan patients presenting *FBN1* mutations are more likely to develop MVP than those having alterations in *TGFBR2* gene (i.e., 45% vs. 21%) (27, 56).

Indeed, LTBPs decrease TGF-β signaling by modulating its interaction with the corresponding receptors (33). When fibrillin is mutated, such modulating function is missing and TGF-β is overexpressed, thereby increasing the activation of its signaling pathway. This leads to the secretion of ECM proteins and to the proliferation of valvular interstitial cells (35, 57, 58). As a matter of fact, there is strong evidence suggesting that excessive TGF-β signaling leads to many features of Marfan syndrome.

Mouse models with *FBN1* mutations were found to develop longer and thicker MV leaflets than wild-type ones, secondarily to TGF-β pathway upregulation (35, 56). Moreover, TGF-β signaling antagonization has been shown to prevent the pathological prolongation and thickening of MV components (35). Indeed, mouse models of Marfan syndrome have been shown to respond positively to TGF-β signaling inhibition, thereby stimulating further investigations on this pathway in human patients. Moreover, proteins belonging to the TGF-β family can also be inhibited by blocking ATR1, a pathway also involved in the pathogenesis of non-syndromic myxomatous MVP, as mentioned above (8, 15, 37, 39, 55, 58). As a matter of fact, Marfan patients have been shown to respond well to losartan (AT1R inhibitor), thus showing reduced aortic root dilatation as compared to controls (8).

### 4.2. Loeys-Dietz syndrome

**Figure 5:**
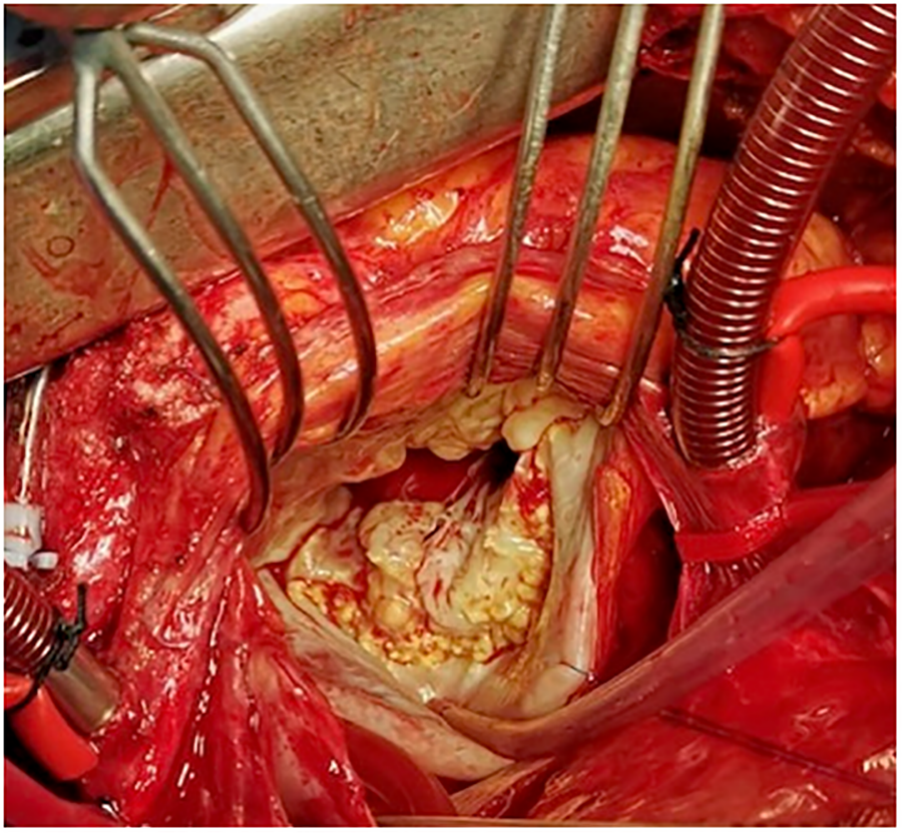
Intraoperative picture of syndromic myxomatous mitral valve prolapse in an 18-year-old boy with Loeys-Dietz syndrome. Leaflets show excessive tissue with impaired elasticity and thin fibrotic deposits, especially in proximity to the annulus.

Loeys-Dietz syndrome is a rare autosomal dominant CTD. Affected individuals often have a combination of skeletal malformations including craniosynostosis and scoliosis, hypertelorism and cleft palate, either pectus excavatum or carinatum, clubfoot and pes planus. Loeys-Dietz syndrome is associated with heterozygous mutations in genes encoding the components of TGF-β receptors 1 and 2. Similarly to Marfan syndrome, LDS patients show an excessive TGF-β signaling which favors the development of MVP (59). Indeed, MVP has been observed in 29% of patients in the case series analyzed by Loeys et al. (Figure 5) (59). Although generally mutations in TGF-β receptors should decrease its downstream signaling, in LDS the direct effect is a nuclear accumulation of phosphorylated SMAD2 and increased connective tissue growth factor (59).

A similar condition to LDS is represented by the aneurysms-osteoarthritis syndrome, characterized by aortic aneurysms, arterial tortuosity, craniofacial abnormalities, and osteoarthritis. This condition is associated with an inactivating mutation in the mother against decapentaplegic homologue 3 (*MADH3*) gene, encoding for the SMAD3, a regulator of TGF-β signaling that promotes TGF-β overexpression. In this condition, MVP occurrence has been reported in up to 50% of patients (60).

### 4.3. Ehlers-Danlos syndrome

Ehlers-Danlos syndrome includes a heterogeneous group of inherited disorders presenting mutations in collagen I, III, V, or XI genes, and affecting connective tissues, primarily at the level of the skin, joints, and blood vessel walls. The overall prevalence of EDS ranges between 1 in 3,500 and 1 in 5,000 people (61). The commonest clinical phenotypes recognized include joint hypermobility, tissue fragility and skin hyperextensibility (61). Currently, up to thirteen subtypes of this syndrome have been identified. The hypermobile variant of EDS (hEDS) is the commonest; however, a rarer subtype, defined as cardiac-valvular EDS (cvEDS), is more often associated with MVP, skin problems and joint hypermobility (62). The cvEDS variant is of autosomal recessive origin and is caused by mutations in the collagen type 1 α2 chain (*COL1A2*) gene (63–66).

The mechanism underlying MVP in EDS patients is not well understood yet, but the main genetic alterations associated with this condition are known to cause a defect in the structure and processing of collagen fibers. It has been speculated that such defects decrease the stiffness and increase the flexibility of connective tissue, resulting in an excessive biomechanical tension within the valve tissue during heart systole (11). The increased biomechanical signaling resulting from such excessive tension may stimulate intracellular signaling at the level of valvular interstitial cells, therefore increasing the production of glycosaminoglycans and other ECM components that are associated with MVP development (67).

### 4.4. Pseudoxanthoma elasticum

Pseudoxanthoma elasticum is a rare CTD affecting approximately 1 in 50,000 people worldwide. This condition is caused by mutations in ATP-binding cassette subfamily C member 6 (*ABCC6*) gene that leads to an absent or nonfunctional MRP6 protein. The result is an accumulation of deposits of calcium and other minerals (i.e., mineralization) in the elastic fibers (68). Given the role of the elastic fibers component of connective tissue in providing strength and flexibility to body structures, these patients typically present a diffuse loss of elasticity. Moreover, PXE patients develop early atherosclerotic calcifications affecting already dystrophic elastic fibers, with loss of vessel wall elasticity, also involving the coronary arteries, and MVP. Therefore, such genetic and histological backgrounds are significantly different from the other syndromes presented and, when present, identify a peculiar form of MV disease (23).

In this population, MVP has been traditionally identified in more than 50% of patients, as reported by the most representative studies in the 80s, when the diagnosis of MVP lied upon different bases with respect to the modern echocardiographic era (69). As a matter of fact, recent reports showed that in PXE patients MVP occurrence rate is about 4.5%, which is not significantly higher than the general population, although the histopathological findings are characteristic of the underlying alterations inherited in this syndrome (23). Although its specific pathophysiological mechanism is unknown, it is hypothesized that an abnormal degeneration of collagen fibers and loss of elastin with fibrous thickening of the endocardium represent the main cause of MV disease, also involving the left ventricle and leading to a sort of restrictive cardiomyopathy with subendocardial calcific deposits (23, 68, 69).

### 4.5. Filamin A mutation syndrome

Filamin A mutation syndrome is an X-linked form of myxomatous valvular dystrophy affecting all the four cardiac valves. Filamin A is a protein encoded by the *FLNA* gene present on chromosome Xq28 (15). It is an actin-binding protein serving to stabilize cortical F-actin networks and linking them to cell membranes, thereby providing membrane integrity and protection against mechanical stress.

Filamin A binds to various cellular proteins, including trans-membrane receptors and signaling molecules, playing a significant role for embryological development, cell migration and mechanical stress response. N-terminal filamin A mutations associated with valvular dystrophy are also involved in small Rho-GTPases regulation, making the affected cells less capable of spreading and migrating on culture surfaces. Indeed, mutated filamin A alters the balance between RhoA and Rac1 GTPases in favor of the first one, therefore impairing the role of Rac1-specific Rho GTPase activating protein 24 (also known as FilGAP) and the downstream trafficking of β1-integrins to the cell membrane (32).

A complete loss of filamin A leads to fetal death due to major malformations involving all the heart and vascular components. Indeed, filamin A has an essential role in intercellular junctions and its mutations influence various signaling pathways involved in valve growth modulation, potentially leading to excessive TGF-β and 5-hydroxytryptamine activation, ultimately causing valvular defects (4, 70, 71).

### 4.6. Arrhythmogenic mitral valve prolapse

In a subgroup of patients, MVP has also been associated with an increased risk of malignant ventricular arrhythmias and sudden cardiac death (SCD), which in fact has been observed in 0.4%–1.8% of cases (72, 73). The arrhythmogenic mechanism is attributed to fibrotic changes occurring on the MV leaflets secondary to glycosaminoglycans accumulation, ventricular friction lesions and excessive traction on papillary muscles caused by pathological systolic shortening (74). Various reports have shown that MVP can induce an increased tension on MV annulus, chordae tendineae and papillary muscles, therefore contributing to the progressive development of myocardial fibrosis, in a complex interplay between the MV and left ventricle (11, 75–79). It is still to be elucidated whether fibrosis represents a consequence of MVP or it is related to genetic predisposition (or both), but in this group of patients myocardial fibrosis and inflammation have been identified in the regions burdened by higher mechanical strain. As a result, fibrotic areas represent potentially arrhythmogenic foci (79). Recent studies have shown the presence of myocardial fibrosis in mouse models of MVP with *DZIP1* mutations (79).

This subset of MVP patients at higher risk of SCD shows also electrocardiographic features, such as ST-segment depression, T wave inversion or biphasic T waves in inferior leads, and premature ventricular contractions arising from the papillary muscles and the fascicular system (80, 81).

Mitral annular disjunction represents another feature characterizing arrhythmogenic MVP syndrome, whereby through echocardiography it is possible to recognize an anatomical “detachment” of the MV annulus from the ventricular myocardium. Although such alteration is more commonly observed under P1 and P2, it involves the whole MV annular circumference, just sparing the mitro-aortic curtain. Mitral annular disjunction has been observed to be associated with arrhythmic events and premature ventricular contractions, with a negative prognostic impact on the affected MVP patients (74).

## 5. Clinical perspectives

Although the large amount of research conducted during the years in the field of molecular biology of MVP, mostly based on animal studies, the application of those findings in daily clinical practice is still limited. However, interesting areas of future development with potential clinical impact can be identified. Indeed, the information about genetic and molecular patterns gained for familial clusters of non-syndromic MVP, as well as the more consistent data available for syndromic forms, might provide enough robustness to identify potential risk profiles of MR development and progression and therefore delineate dedicated follow-up protocols based on prespecified biomarkers (9, 79). Large scale genetic studies enrolling MVP patients could also be useful to explore and better understand genetic alterations, regulation and expression in this condition (79).

The multiple molecular pathways involved in the development of MVP, mostly leading to an unfavorable remodeling of the ECM, could represent potential pharmacological targets able to prevent development or stop progression of MV disease in high-risk patients and would therefore deserve dedicated pre-clinical and clinical studies (29).

Concerning the pharmacological targets of MVP, they represent a promising area of improvement with potentially favorable impact in clinical practice. For instance, ATR1 inhibition has shown promising results to significantly slow the rate of aortic root enlargement and, therefore, delay the need for surgery. Such results have been more effective among patients with *FBN1* mutation (82). Since MVP represents another progressive condition often occurring in Marfan patients, especially when carrying *FBN1* mutation with respect to *TGFBR2* mutation, the potential impact of angiotensin receptor inhibition on MVP development and progression would represent an interesting area of investigation of dedicated studies.

Finally, given the critical role of TGF-β signaling in the development of myxomatous MVP demonstrated also in animal models, further dedicated pre-clinical and clinical studies are advocated to better investigate the impact of the inhibition of this pathway in the pathogenesis and progression of MVP in humans.

## 6. Conclusions

Mitral valve prolapse represents the commonest cause of severe primary MR and the first indication for MV surgery in the developed world. It is a heterogeneous disorder presenting with variable phenotypes and clinical characteristics. The biological mechanisms underlying this condition have been thoroughly investigated for decades and multiple pathways have been identified, mostly focusing on the central role of VICs, TGF-β upregulation and ECM remodeling. A few of these pathways have been also experimentally found to respond well to pharmacological inhibition in animal models (e.g., ATR1 inhibition) and may represent potential targets to limit MR progression. However, no biomarker has still been identified or validated for MVP.

The genetic basis of MVP represents a critical starting point in the investigation of syndromic forms of MVP, especially those accompanying CTDs, as well as the heterogeneous group of familial clusters of non-syndromic MVP. Such investigations have allowed a deeper understanding of the molecular mechanisms underlying this condition, potentially providing useful information to stratify patients according to their risk of MVP development and progression.

Although studies merely on animal models and genome-wide analyses have strongly supported the scientific progression in our knowledge of this disorder, further studies are advocated to improve our understanding of MVP predisposition, to possibly identify some specific biomarkers and pathogenetic pharmacological targets, therefore providing a complementary aid to interventional treatment that yet remains the only curative option for these patients.
